# Nomogram for Predicting Recurrence-Free Survival of Primary Localized Gastrointestinal Stromal Tumor

**DOI:** 10.3390/jpm13030498

**Published:** 2023-03-10

**Authors:** Pan Ran, Tao Tan, Hui Zhou, Jinjin Li, Hao Yang, Juan Li, Jun Zhang

**Affiliations:** 1 Department of Gastrointestinal Surgery, the First Affiliated Hospital of Chongqing Medical University, Chongqing 400016, China; 2 Department of Internal Medicine, Chongqing Key Laboratory of Translation Research for Cancer Metastasis and Individualized Treatment, Chongqing University Cancer Hospital, Chongqing 400030, China; 3 Department of Pharmacy, the First Affiliated Hospital of Chongqing Medical University, Chongqing 400016, China

**Keywords:** recurrence-free survival, gastrointestinal stromal tumor, nomogram, individualized treatment, prediction

## Abstract

Purpose: This study aimed to establish a new nomogram that predicts recurrence-free survival (RFS) after a complete surgical resection of primary localized gastrointestinal stromal tumors (GISTs); it also aimed to evaluate the discrimination, calibration, and clinical utility of the decision-making nomogram. Methods: The clinicopathological data of patients with primary localized GISTs at the First Affiliated Hospital of Chongqing Medical University from January 2000 to June 2022 were retrospectively analyzed. The clinicopathological data were randomly split into two sets (7:3 ratio) for training and validation. Suitable variables for the construction of a nomogram for the 1-, 3-, and 5-year RFS were selected using univariate and multivariate Cox regression analyses. Receiver operating characteristic (ROC) analysis and a concordance index (C-index) were used to quantify the discrimination of the nomogram and were compared with four commonly used prognostic scoring systems: Memorial Sloan Kettering Cancer Center prognostic nomogram, National Institutes of Health–Fletcher staging system, Chen’s prognostic nomogram, and Air Forces Institute of Pathology risk criteria–Miettinen staging system. The calibration and clinical utility for the decision-making nomogram were validated using calibration curves and decision curves, respectively. Results: In total, 641 patients were screened and analyzed in this retrospective, observational study. RFS was significantly related to tumor size, mitotic count, gender, DOG-1, and adjuvant therapy with imatinib according to the results of the multivariate and univariate Cox analyses. The nomogram was constructed using the above variables (all *p* < 0.05) for the 1-, 3-, and 5-year RFS. In the training set, the 1-, 3-, and 5-year ROC and C-index values of the nomogram were 0.868, 0.838, 0.816, and 0.830, respectively. For internal validation, we performed model fitting on the validation set, and the 1-, 3-, and 5-year ROC and C-indices were 0.977, 0.845, 0.869, and 0.849, respectively. Among the five GIST prognostic scoring systems, our nomogram had almost all the largest area under these decision curves and had a good calibration capability. Conclusions: The newly constructed nomogram based on tumor size, gender, mitotic count, DOG-1, and adjuvant treatment with imatinib exhibited an excellent performance and may serve as a prognostic scoring system to support therapeutic decision-making and individualized treatment for GISTs in China.

## 1. Introduction

Gastrointestinal stromal tumors (GISTs) are the most common mesenchymal tumors of the digestive tract [1,2]. The key way of differentiating GISTs from other stromal tumors is the expression of CD117, DOG-1, and CD34, of which the positivity of DOG-1 may be a potentially ideal diagnostic biomarker [3]. They can occur anywhere throughout the digestive tract, and on rare occasions, outside of the tract, such as in the retroperitoneal space, omentum, and mesentery [4]. The only possible curative treatment for primary localized GISTs is surgical resection [5]. However, even after a complete surgical resection, within 5 years, a substantial number of patients with primary localized GISTs experience tumor recurrence with local or distant metastasis, which can eventually lead to death [6,7].

In 2002, Fletcher offered a consensus method based on two pathological indices: tumor size and mitotic count [8]. Then, 4 years later, Miettinen proposed the Armed Forces Institute of Pathology (AFIP) criteria according to the clinicopathologic data of 1939 GIST patients from previously published long-term follow-up studies, which included tumor site as an important risk factor in addition to tumor size and mitotic count [3]. An initial prognostic nomogram was developed by Memorial Sloan Kettering Cancer Center (MSKCC) to predict the likelihood of recurrence-free survival (RFS) after the resection of primary localized GISTs in 2009 [9]. Unfortunately, a limitation of the above studies is the lack of data from patients in Asia. In 2018, Chen et al. used data from Chinese patients to develop a new prognostic nomogram, which was the first to include the Ki-67 labeling index (Li) in the construction of the nomogram [10]. However, in this study, tumor size and the Ki-67 Li were included as dichotomous variables for the construction of the model, which may affect the model’s performance.

Accordingly, this study aimed to establish a new nomogram for predicting the RFS of patients with primary localized GISTs. Additionally, we compared the predictive performance and clinical practicability of our new nomogram with those of the MSKCC prognostic nomogram, NIH–Fletcher staging system, Chen’s prognostic nomogram, and AFIP–Miettinen staging system to assess its clinical value.

## 2. Materials and Methods

### 2.1. Patients

We retrospectively analyzed the clinicopathological data of patients with GISTs who were followed up at the First Affiliated Hospital of Chongqing Medical University from January 2000 to June 2022. The patient follow-up was concluded on 1 September 2022. The inclusion criteria were as follows: (1) patients with primary localized GISTs underwent surgical resection with curative intent; (2) patients had less than 15% missing data; and (3) in the adjuvant or neoadjuvant setting, patients did not receive any other tyrosine kinase inhibitors other than imatinib. The exclusion criteria were as follows: (1) age at diagnosis < 18 years; (2) patients had a history of chemoradiation; (3) tumor ruptured before or during the operation; (4) follow-up time ≤ 3 months; and (5) patients had a history of any malignancies.

**Figure 1:**
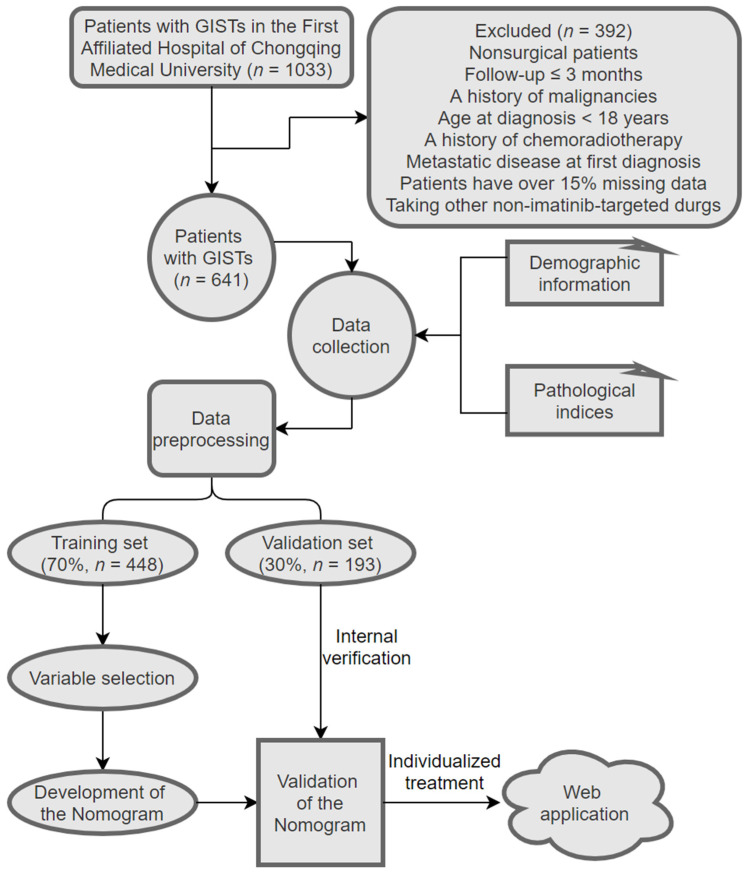
Patient selection process and study design.

With the help of the website “Extreme Smart Analysis” (https://www.xsmartanalysis.com/, accessed on 22 February 2023), patients with GISTs were randomly divided into two groups according to a ratio of 7:3: the training set (70%) for developing the nomogram and the validation set (30%) for an evaluation of the performance. By contrasting the baseline characteristics and RFS between the training and validation sets, the success of randomization was checked. The inclusion and exclusion criteria and the schematic representation of the study design are displayed in Figure 1.

### 2.2. Collection of Demographic, Pathological, and Follow-Up Data

All the demographic information and pathological indices were collected from the “Weinichangzai” database. The demographic information included the age at diagnosis, gender, residence, initial symptoms, and treatment history. Pathological information included the tumor size, Ki-67 Li, mitotic count, tumor site, gene mutation, and expression of DOG-1/CD117/CD34. Missing data were supplemented using the random forest algorithm [11,12].

Follow-up information was collected from the GIST specialist outpatient clinic, telephone calls, WeChat, and other interaction tools every 3–6 months until the patient’s death or until they were lost to follow-up. The appearance of new lesions on computed tomography or abdominal ultrasonography was defined as tumor recurrence. RFS was used as the endpoint, and RFS was defined as the time from complete surgical resection to a recurrence or last contact.

### 2.3. Statistical Analysis

The normal distribution of the variables was assessed by a histogram and Kolmogorov–Smirnov tests. Continuous variables were described using the median or interquartile range (IQR) for non-normal distribution variables, and the mean ± standard deviation (SD) was used for normal distribution variables. The Mann–Whitney U-test (non-normal distribution variables) and Student’s *t*-test (normal distribution variables) were used to assess the differences in the continuous variables between the training and validation sets. Categorical variables were presented with frequencies (percentages). Pearson′s chi-squared test and Fisher′s exact test were used to assess the differences in the categorical variables between the training and validation sets. Statistical significance was set at *p* < 0.05; all *p* values were 2-tailed; and all statistical analyses were performed using R software (R 4.2.1, R Core Team, 2022).

### 2.4. Evaluation of the Nomogram Model’s Performance

The predictive value of the decision-making nomogram was evaluated based on its model discrimination, model calibration, and clinical utility. We assessed the discrimination of the model in the training set using receiver operator characteristic (ROC) analysis at 1-, 3-, and 5-year marks, as well as the Harrell concordance index (C-index). We then assessed the clinical utility of the decision-making nomogram in the training set using decision curve analysis (DCA) at 1-, 3-, and 5-year points. For internal validation, we performed model fitting on the validation set. Finally, we compared the area under the curve (AUC) of the ROC, C-index, and DCA with those of other models: the MSKCC prognostic nomogram [9], NIH–Fletcher staging system [8], Chen’s prognostic nomogram [10], and AFIP–Miettinen staging system [3]. The calibration ability of the model was confirmed through an internal validation using bootstrap sampling with 200 random samples and a validation set, and this was described by a calibration curve at 1-, 3-, and 5-year marks.

## 3. Results

### 3.1. Patient Characteristics

Table 1 displays the baseline demographic information and pathological information of the 641 patients (360 female and 281 male) with primary localized GISTs. The median duration of the follow-up was 144.40 months. The average age at diagnosis of confirmed GIST patients was 55.60 ± 11.91 years. The median tumor size and Ki-67 Li were 6 cm (4–9 cm) and 5% (3–10%), respectively. Most patients were from cities (70.20%; 450 patients), but 191 (29.80%) were from rural townships. There were 443 patients (69.11%) with mitotic counts ≤ 5 per/50 HPF, 138 patients (21.53%) with mitotic counts less than or equal to 10 but more than 5 per/50 HPF, and 60 patients (9.36%) with mitotic counts > 10 per/50 HPF. Among the 641 patients, the most common gene mutation was the KIT exon 11 mutation (69.58%), followed by the KIT exon 9 mutation (17.16%), PDGFR-α (3.28%), wild type (8.74%), and other gene mutations (1.25%). The numbers in parentheses represent the percent of patients in the respective category; they may not add up to 100% due to rounding.

**Figure 2:**
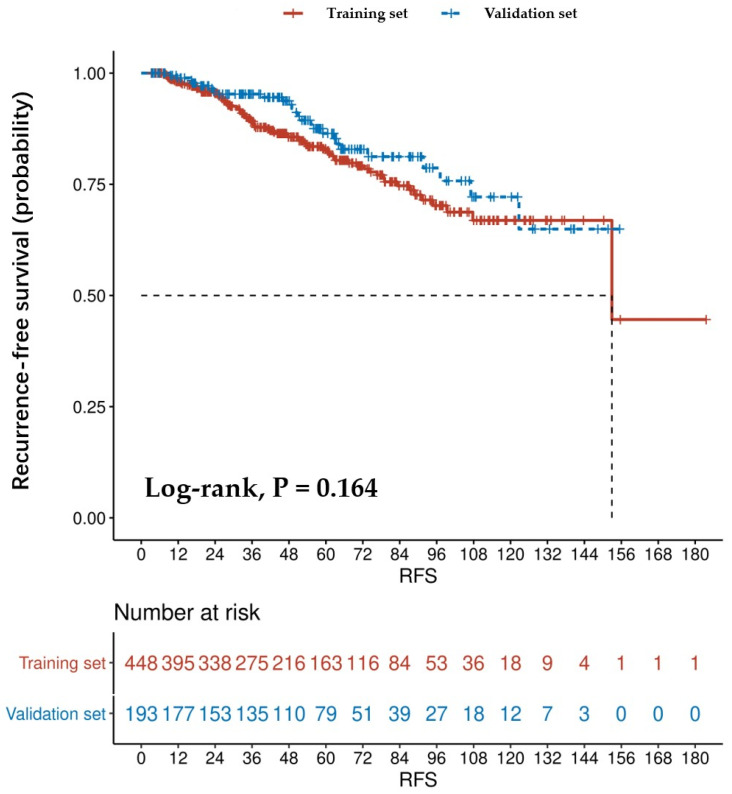
RFS curves of training (*n* = 448) and validation (*n* = 193) sets who underwent surgical resection.

We used Kaplan–Meier curves to compare RFS in patients in the training and validation sets (Figure 2). The RFS rates in the training set after 1, 3, and 5 years were 88.17%, 61.38%, and 36.38%, respectively. Additionally, the RFS rates in the validation set after 1, 3, and 5 years were 91.71%, 69.95%, and 40.93%, respectively.

### 3.2. Creation of the Nomogram

**Figure 3:**
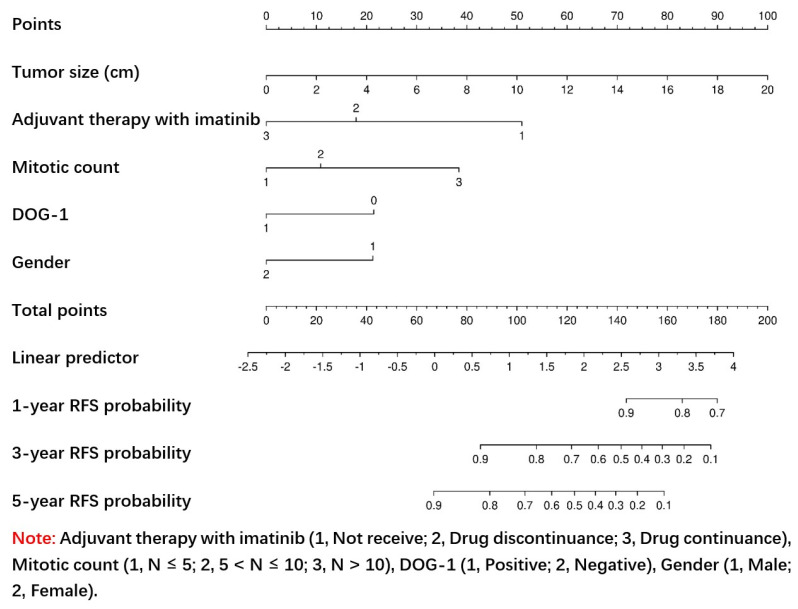
Nomogram predicting the probabilities of 1-, 3-, and 5-year RFS. Points are assigned for tumor size, gender, mitotic count, DOG-1, and adjuvant therapy with imatinib by drawing a line upward from the corresponding values to the “Points” line. The sum of these five points plotted on the “Total Points” line corresponds to predictions of 1-, 3-, and 5-year RFS.

This study used univariate and multivariate Cox regression analysis to identify the factors associated with RFS within the training set (*p* < 0.05). Univariate Cox regression analysis showed that the tumor size, Ki-67 Li, gender, initial symptom, mitotic count, tumor site, DOG-1, and adjuvant therapy with imatinib were significant predictors of RFS (*p* < 0.05; Table 2). To identify independent prognostic factors, all significant variables in the univariate Cox regression analysis (*p* < 0.05) were subjected to multivariate Cox regression analysis. Multivariate Cox regression analysis showed that the tumor size, gender, mitotic count, DOG-1, and adjuvant therapy with imatinib were significant predictors of RFS (*p* < 0.05; Table 2). From the univariate and multivariate Cox regression results within the training set, a nomogram for the 1-, 3-, and 5-year RFS was constructed (Figure 3). By calculating the scores for each variable and projecting the total scores onto the bottom scale, the probabilities of 1-, 3-, and 5-year RFS were predicted. An online tool based on the formulated nomogram is available at https://weinichangzai.shinyapps.io/My_COX/ (accessed on 22 February 2023), which creates an interactive web application for individualized patient prediction. Patients with GISTs can easily access this website to construct prognostic predictions accordingly.

### 3.3. Evaluation of the Accuracy and Clinical Utility of Nomogram

**Figure 4:**
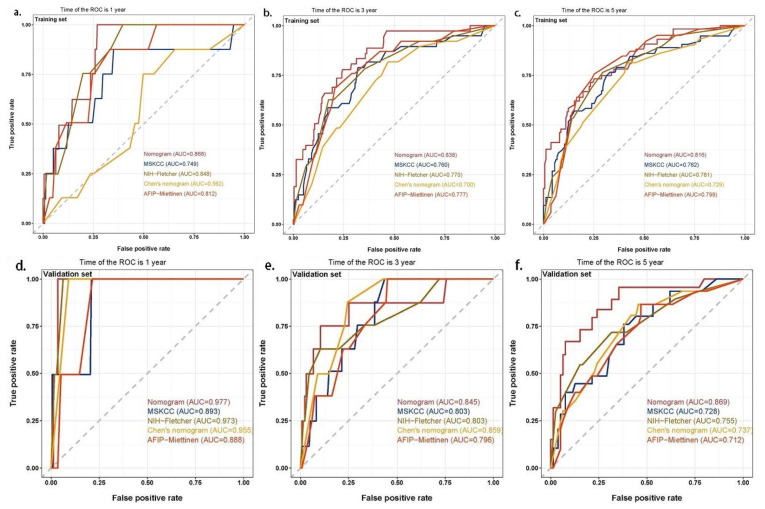
ROC curves for each prognostic scoring system to predict the RFS of patients with GISTs. (**a**) The 1-year ROC curves in the training set. (**b**) The 3-year ROC curves in the training set. (**c**) The 5-year ROC curves in the training set. (**d**) The 1-year ROC curves in the validation set. (**e**) The 3-year ROC curves in the validation set. (**f**) The 5-year ROC curves in the validation set.

The ROC and C-index were used to quantify the model discrimination of the nomogram and they were compared with four commonly used prognostic scoring systems: the MSKCC prognostic nomogram, NIH–Fletcher staging system, Chen’s prognostic nomogram, and AFIP–Miettinen staging system. According to the results, the 1-, 3-, and 5-year AUC and C-index in the training set were as follows: nomogram, 0.868, 0.838, 0.816, and 0.830; MSKCC prognostic nomogram, 0.749, 0.760, 0.762, and 0.747; NIH–Fletcher staging system, 0.848, 0.770, 0.781, and 0.763; Chen’s prognostic nomogram, 0.562, 0.700, 0.729, and 0.702; and AFIP–Miettinen staging system, 0.812, 0.777, 0.799, and 0.763, respectively. For the internal validation, we performed model fitting on the validation set. In light of the findings, the 1-, 3-, and 5-year AUC and C-index in the validation set were as follows: nomogram, 0.977, 0.845, 0.869, and 0.849; MSKCC prognostic nomogram, 0.893, 0.803, 0.728, and 0.745; NIH–Fletcher staging system, 0.973, 0.803, 0.755, and 0.766; Chen’s prognostic nomogram, 0.955, 0.859, 0.737, and 0.768; and AFIP–Miettinen staging system, 0.888, 0.796, 0.712, and 0.751, respectively. The above results showed that the nomogram’s model discrimination was superior to that of other GIST prognostic scoring systems (Table 3 and Figure 4).

**Figure 5:**
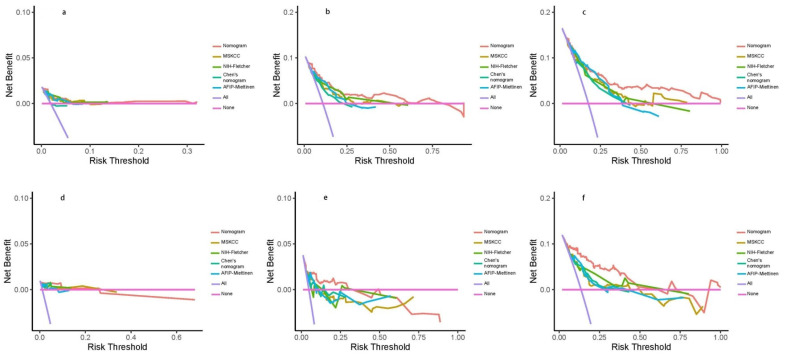
The DCA for each prognostic scoring system. (**a**) The 1-year DCA curves in the training set. (**b**) The 3-year DCA curves in the training set. (**c**) The 5-year DCA curves in the training set. (**d**) The 1-year DCA curves in the validation set. (**e**) The 3-year DCA curves in the validation set. (**f**) The 5-year DCA curves in the validation set.

To compare the prediction models regarding their clinical utility of the training and validation sets, DCA was used. As shown in Figure 5, among the five GIST prognostic scoring systems, the area under the decision curve of the nomogram model was almost the largest compared to the other four models.

### 3.4. Confirmation of the Nomogram

**Figure 6:**
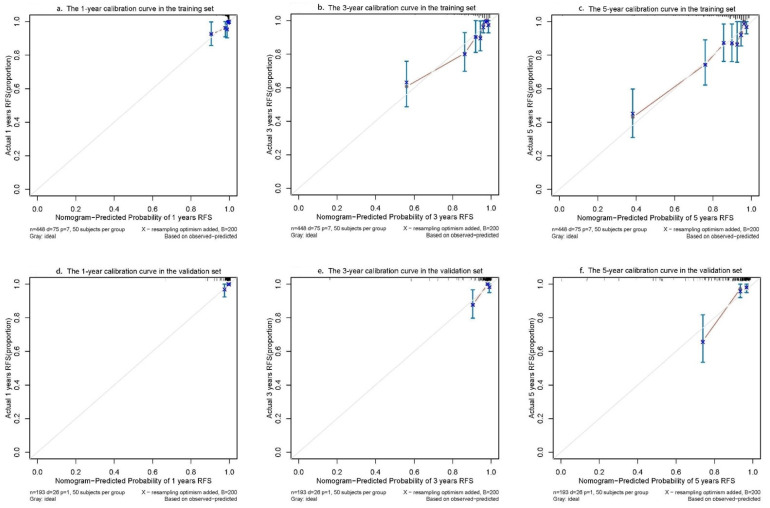
Calibration plots for the training and validation sets that show the predicted and observed RFS rates. (**a**) The 1-year calibration curve in the training set. (**b**) The 3-year calibration curve in the training set. (**c**) The 5-year calibration curve in the training set. (**d**) The 1-year calibration curve in the validation set. (**e**) The 3-year calibration curve in the validation set. (**f**) The 5-year calibration curve in the validation set.

As shown in Figure 6, calibration curves at 1, 3, and 5 years were used to assess the discrimination ability of the model by plotting the actual RFS against the nomogram-predicted probability of RFS, where a smaller distance from the scatter points to the 45° line indicated a better calibration ability. The calibration curve demonstrated that the calibration in the training and validation sets was accurate (Figure 6). The results demonstrated that the nomogram was capable of accurately predicting the RFS of patients with primary localized GISTs who underwent surgical resection.

## 4. Discussion

This study describes the accuracy, clinical utility, and discriminatory ability of a prognostic nomogram to predict RFS following a complete surgical resection of primary localized GISTs. A nomogram that assigns predictions for 1-, 3-, and 5-year RFS based on the tumor size, gender, mitotic count, DOG-1, and adjuvant therapy with imatinib was created based on clinicopathological data from 641 patients from the First Affiliated Hospital of Chongqing Medical University.

Some researchers consider that reasonable screening variables can improve the risk prediction probability of the current prognostic scoring systems [13,14]. In this study, based on the results of the multivariate Cox regression analysis, a new nomogram was constructed that integrates multiple predictors and expresses the relationship between each variable in the prediction model using line segments with scales. In practice, clinicians are able to quickly predict outcomes because of the nomogram’s straightforward graphical portrayal [15,16]. The nomogram in this study showed the best model performance compared with other prognostic scoring systems, including the accuracy, clinical utility, and discrimination ability.

In the above five models, the tumor size and mitotic count were two important factors in the construction of the model. Likewise, several studies have confirmed the importance of the tumor size and mitotic count as prognostic factors [17,18,19]. Few studies have used gender and DOG-1 as independent prognostic factors for GISTs [20,21]. Some scholars concluded that the negative expression of DOG-1 may be predictive of the malignant outcome of patients with GISTs, and it is significantly associated with a shorter period of overall survival [21,22]. However, further research suggested that the expression of DOG-1, CD117, and CD34 were usually selected as diagnostic biomarkers, but not prognostic biomarkers [3,23].

Whether gender is an independent prognostic factor for RFS or not is still a controversial issue. The results of the current study are consistent with those of previous studies conducted by Zhang et al. [17] and Sun et al. [24] that concluded that the male sex may be associated with poorer RFS outcomes. It was also reported that the male gender was a significantly unfavorable prognostic factor in Chinese patients with GISTs [25]. However, in some previous clinical trials, gender was not considered to be an independent prognostic factor for primary localized GISTs, at least not at a physiological level [26,27]. To our knowledge, this is the first study to incorporate gender and DOG-1 as predictive factors, which may reflect differences in the patients’ demographics. Therefore, the findings of this study may apply to Chinese patients with GISTs.

In addition, adjuvant therapy with imatinib was significantly associated with RFS. For primary localized GISTs, the first-line adjuvant treatment is imatinib, a tyrosine kinase inhibitor [28]. Other retrospective and prospective trials have confirmed that imatinib can minimize tumor recurrence [29]. Unfortunately, confirmation of the final timing of the use of an adjuvant therapy with imatinib is pending the results of ongoing controlled studies [30,31]. However, it is interesting to note that in the later stages of treatment, the remaining variables (tumor size, mitotic count, gender, and DOG-1) used to construct the nomogram could not be changed, with the exception of the variable of an adjuvant therapy with imatinib. Based on this, patients with GISTs with a higher score summation of other variables (tumor size, mitotic count, gender, and DOG-1) may be advised to continue taking imatinib to improve prognosis.

For primary localized GISTs, being able to anticipate the possibility of a postoperative recurrence is crucial for various reasons [9]. First, patients can receive proper counseling regarding their possible outcomes. Second, the nomogram may be useful in guiding outpatient service doctors regarding the frequency and intensity of the postoperative follow-up. Most importantly, a 5-year postoperative recurrence is common in GISTs; for example, in the training and validation sets, the 5-year recurrence rates were 63.62% and 59.07%, respectively (Figure 2).

This study had some limitations that should be addressed and rectified in future research. First, our study included a small sample and it was a single-center study. Therefore, to confirm the predictive performance of this nomogram in the future, multicenter studies are required. Second, as this was a retrospective study with a long study period, it is subject to all the limitations associated with retrospective studies. Third, patients with GISTs who did not receive an adjuvant therapy with imatinib or who discontinued the adjuvant therapy with imatinib were also included, so medication adherence was not included in this study. Finally, previous clinical studies have confirmed that some indices in preoperative general blood tests are associated with the prognosis of patients with GISTs [16,32,33,34,35,36]. Therefore, our findings may have been more conclusive if we included the data of preoperative general blood tests in our analysis. Considering the various limitations of our study, further research is needed in order to supplement our research.

In addition to the limitations mentioned above, this study had several advantages in its design. All factors constructed in the nomogram to predict the RFS of patients with GISTs are easy to obtain in routine clinical practice, which further increases the utility of our model. In addition, the newly constructed nomogram had a high discrimination level and calibration performance for predicting the RFS of patients with primary localized GISTs in the training and validation sets. Finally, to achieve the goal of an individualized treatment for GIST, a web-based interface was provided for an easy prediction.

## 5. Conclusions

The RFS of patients with primary localized GISTs was predicted in this study using a nomogram based on the tumor size, gender, mitotic count, DOG-1, and an adjuvant treatment with imatinib. The finding that the nomogram could provide an accurate prediction of recurrence risk for individual patients with GISTs was validated in an independent validation set. Additionally, the nomogram can help physicians in our center provide individualized treatment and monitoring programs for patients with GISTs.

## Figures and Tables

**Table 1:** Baseline characteristics of GIST patients in the training and validation sets.

Variable	Total	Training Set	Validation Set	*p* Value
	*(n* = 641)	(*n* = 448)	(*n* = 193)	
Age at diagnosis (years)	55.60 ± 11.91	55.67 ± 11.89	55.44 ± 12.00	0.827 ^a^
Tumor size (cm)	6 (4–9)	6 (4–9)	6 (4–9)	0.757 ^b^
Ki-67 Li (%)	5 (3–10)	5 (3–10)	5 (3–10)	0.953 ^b^
Gender				0.556 ^c^
Male	281 (43.84%)	193 (43.08%)	88 (45.60%)	
Female	360 (56.16%)	255 (56.92%)	105 (54.40%)	
Residence				0.776 ^c^
City	450 (70.20%)	313 (69.87%)	137 (70.98%)	
Rural township	191 (29.80%)	135 (30.13%)	56 (29.02%)	
Initial symptom				0.333 ^d^
Asymptomatic	199 (31.05%)	139 (31.03%)	60 (31.09%)	
Hematemesis	15 (2.34%)	9 (2.01%)	6 (3.11%)	
Hematochezia	141 (22.00%)	92 (20.54%)	49 (25.39%)	
Abdominal discomfort	263 (41.03%)	194 (43.30%)	69 (35.75%)	
Sour regurgitation	12 (1.87%)	8 (1.79%)	4 (2.07%)	
Eating obstruction	11 (1.72%)	6 (1.34%)	5 (2.59%)	
Mitotic count (per 50 HPFs)				0.278 ^c^
N ≤ 5	443 (69.11%)	315 (70.31%)	128 (66.32%)	
5 < N ≤ 10	138 (21.53%)	89 (19.87%)	49 (25.39%)	
N > 10	60 (9.36%)	44 (9.82%)	16 (8.29%)	
Tumor site				0.290 ^c^
Stomach	323 (50.39%)	222 (49.55%)	101 (52.33%)	
Small intestine	249 (38.85%)	183 (40.85%)	66 (34.20%)	
Colorectum/rectal	31 (4.84%)	20 (4.46%)	11 (5.70%)	
Other	38 (5.93%)	23 (5.13%)	15 (7.77%)	
CD34				0.091 ^c^
Negative	74 (11.54%)	58 (12.95%)	16 (8.29%)	
Positive	567 (88.46%)	390 (87.05%)	177 (91.71%)	
CD117				0.972 ^c^
Negative	23 (3.59%)	16 (3.57%)	7 (3.63%)	
Positive	618 (96.41%)	432 (96.43%)	186 (96.37%)	
DOG-1				0.994 ^c^
Negative	59 (9.20%)	41 (9.15%)	18 (9.33%)	
Positive	582 (90.80%)	407 (90.85%)	175 (90.67%)	
Gene mutation				0.159 ^d^
KIT exon 11	446 (69.58%)	301 (67.19%)	145 (75.13%)	
KIT exon 9	110 (17.16%)	87 (19.42%)	23 (11.92%)	
PDGFR-α	21 (3.28%)	16 (3.57%)	5 (2.59%)	
Wild type	56 (8.74%)	39 (8.71%)	17 (8.81%)	
Other	8 (1.25%)	5 (1.12%)	3 (1.55%)	
Neoadjuvant therapy with imatinib				0.735 ^c^
Not received	591 (92.20%)	412 (91.96%)	179 (92.75%)	
Received	50 (7.80%)	36 (8.04%)	14(7.25%)	
Adjuvant therapy with imatinib				0.929 ^c^
Not received	171 (26.68%)	118 (26.34%)	53 (27.46%)	
Drug discontinuance	104 (16.22%)	72 (16.07%)	32 (16.58%)	
Drug continuance	366 (57.10%)	258 (57.59%)	108 (55.96%)	

Note: ^a^ Student’s t-test; ^b^ Mann–Whitney U-test; ^c^ chi-squared test; ^d^ Fisher’s test. We calculated all *p* values as two-tailed. Age at diagnosis refers to the age at diagnosis of primary localized GIST. Abbreviations: DOG-1, gastrointestinal stromal tumor protein 1; CD117, cluster of differentiation 117; CD34, cell differentiation factor 34; HPF, high-power field.

**Table 2:** Univariate and multivariate Cox analysis of prognostic variables in the training set for the RFS of patients with GISTs.

Variable	RFS			
	Univariate		Multivariate	
	HR (95% CI)	*p* Value	HR (95% CI)	*p* Value
Age at diagnosis (years)	1.00 (0.99–1.02)	0.776	-	
Tumor size (cm)	1.16 (1.11–1.20)	<0.001	1.00 (0.97–1.04)	0.898
Ki-67 Li (%)	1.04 (1.02–1.06)	<0.001	1.12 (1.07–1.18)	<0.001
Gender		0.002		0.024
Male	1.0 (ref)		1.0 (ref)	
Female	0.53 (0.36–0.79)		0.62 (0.41–0.94)	
Residence		0.543	-	
City	1.0 (ref)			
Rural township	1.14 (0.74–1.76)			
Initial symptom		0.016		0.116
Asymptomatic	1.0 (ref)		1.0 (ref)	
Hematemesis	1.21 (0.28–5.17)		1.69 (0.38–7.46)	
Hematochezia	1.06 (0.56–2.03)		1.09 (0.56–2.12)	
Abdominal discomfort	2.23 (1.35–3.71)		1.99 (1.18–3.36)	
Sour regurgitation	0.00 (0.00–1.17)		0.00 (0.00–6.65)	
Eating obstruction	1.94 (0.58–6.52)		1.54 (0.44–5.46)	
Mitotic count (per 50 HPFs)		<0.001		<0.001
N ≤ 5	1.0 (ref)		1.0 (ref)	
5 < N ≤ 10	1.60 (0.99–2.56)		1.53 (0.89–2.65)	
N > 10	5.89 (3.66–9.48)		5.07 (2.66–9.65)	
Tumor site		<0.001		0.417
Stomach	1.0 (ref)		1.0 (ref)	
Small intestine	2.43 (1.57–3.74)		1.51 (0.93–2.46)	
Colorectum/rectal	0.73 (0.18–3.05)		1.28 (0.30–5.45)	
Other	3.20 (1.61–6.34)		1.30 (0.56–3.04)	
CD34		0.357	-	
Negative	1.0 (ref)			
Positive	0.78 (0.46–1.33)			
CD117		0.255	-	
Negative	1.0 (ref)			
Positive	0.59 (0.24–1.46)			
DOG-1		<0.001		0.036
Negative	1.0 (ref)		1.0 (ref)	
Positive	0.35 (0.22–0.55)		0.60 (0.37–0.97)	
Gene mutation		0.975		
KIT exon 11	1.0 (ref)			
KIT exon 9	1.09 (0.67–1.79)			
PDGFR-α	1.38 (0.43–4.40)			
Wild type	1.14 (0.60–2.16)			
Other	0.00 (0.00–8.22)			
Neoadjuvant therapy with imatinib		0.482	-	
Not received	1.0 (ref)			
Received	0.72 (0.29–1.78)			
Adjuvant therapy with imatinib		<0.001		<0.001
Not received	1.0 (ref)		1.0 (ref)	
Drug discontinuance	0.28 (0.16–0.49)		0.29 (0.16–0.51)	
Drug continuance	0.23 (0.14–0.35)		0.16 (0.10–0.25)	

Note: “-” indicates no data. Abbreviation: RFS, recurrence-free survival; HR, hazard ratio; CI, confidence interval; DOG-1, gastrointestinal stromal tumor protein 1; CD117, cluster of differentiation 117; CD34, cell differentiation factor 34; HPF, high-power field.

**Table 3:** The 1-, 3-, and 5-year AUC and C-index according to different criteria.

Criteria	Training Set				Validation Set			
	1-Year AUC	3-Year AUC	5-Year AUC	C-Index	1-Year AUC	3-Year AUC	5-Year AUC	C-Index
Nomogram	0.868	0.838	0.816	0.830	0.977	0.845	0.869	0.849
MSKCC	0.749	0.760	0.762	0.747	0.893	0.803	0.728	0.745
NIH–Fletcher	0.848	0.770	0.781	0.763	0.973	0.803	0.755	0.766
Chen’s nomogram	0.562	0.700	0.729	0.702	0.955	0.859	0.737	0.768
AFIP–Miettinen	0.812	0.777	0.799	0.763	0.888	0.796	0.712	0.751

Abbreviations: MSKCC, Memorial Sloan Kettering Cancer Center; NIH, National Institute of Health; AFIP, Air Forces Institute of Pathology; AUC, area under the curve; C-index, concordance index.

## Data Availability

The datasets used and analyzed during the current study are available from the corresponding author (Jun Zhang) upon reasonable request.

## Abbreviations

RFS, recurrence-free survival; GIST, gastrointestinal stromal tumor; ROC, receiver operating characteristic; C-index, concordance index; MSKCC, Memorial Sloan Kettering Cancer Center; NIH, National Institutes of Health; AFIP, Air Forces Institute of Pathology; Li, labeling index; IQR, interquartile range; SD, standard deviation; DCA, decision curve analysis; AUC, area under curve; CD117, cluster of differentiation 117; DOG-1, gastrointestinal stromal tumors protein 1; CD34, cluster of differentiation 34.
