# Correction: Ran et al. Nomogram for Predicting Recurrence-Free Survival of Primary Localized Gastrointestinal Stromal Tumor. *J. Pers. Med.* 2023, *13*, 498

**DOI:** 10.3390/jpm14080819

**Published:** 2024-07-31

**Authors:** Pan Ran, Tao Tan, Hui Zhou, Jinjin Li, Hao Yang, Juan Li, Jun Zhang

**Affiliations:** 1 Department of Gastrointestinal Surgery, the First Affiliated Hospital of Chongqing Medical University, Chongqing 400016, China; 2 Department of Internal Medicine, Chongqing Key Laboratory of Translation Research for Cancer Metastasis and Individualized Treatment, Chongqing University Cancer Hospital, Chongqing 400030, China; 3 Department of Pharmacy, the First Affiliated Hospital of Chongqing Medical University, Chongqing 400016, China

In the original publication [1], there were mistakes in Table 2 and Figure 3. In Table 2, “Ki-67 Li” and “Tumor size” were written in the wrong positions for each other. At the same time, the authors mistakenly used all the data (training set + validation set) to conduct multivariate Cox analysis, not the training set alone.

In Figure 3, the “Note” was incorrectly labeled as “DOG-1 (1. Positive; 2. Negative)” instead of “DOG-1 (1. Positive; 0. Negative)”.

The corrected Table 2 and Figure 3 appear below. The authors state that these corrections do not significantly impact the overall findings and conclusions of the paper. This correction was approved by the Academic Editor. The original publication has also been updated.

**Table 2:** Univariate and multivariate Cox analysis of prognostic variables in the training set for the RFS of patients with GISTs.

Variable	RFS			
	Univariate		Multivariate	
	HR (95% CI)	*p* Value	HR (95% CI)	*p* Value
Age at diagnosis (years)	0.99 (0.98–1.01)	0.575	-	
Tumor size (cm)	1.19 (1.13–1.25)	<0.001	1.16 (1.09–1.24)	<0.001
Ki-67 Li (%)	1.04 (1.02–1.07)	<0.001	1.01 (0.97–1.05)	0.519
Gender		0.002		0.003
Male	1.0 (ref)		1.0 (ref)	
Female	0.47 (0.30–0.75)		0.49 (0.30–0.78)	
Residence		0.521	-	
City	1.0 (ref)			
Rural township	1.18 (0.71–1.94)			
Initial symptom		0.380	-	
Asymptomatic	1.0 (ref)			
Hematemesis	0.71 (0.10–5.36)			
Hematochezia	0.90 (0.42–1.90)			
Abdominal discomfort	1.60 (0.93–2.78)			
Sour regurgitation	0.00 (0.00–1.94)			
Eating obstruction	1.93 (0.45–8.32)			
Mitotic counts (per 50 HPFs)		<0.001		0.016
N ≤ 5	1.0 (ref)		1.0 (ref)	
5 < N ≤ 10	1.49 (0.84–2.64)		1.30 (0.68–2.49)	
N > 10	4.79 (2.76–8.33)		3.00 (1.39–6.50)	
Tumor site		0.002		0.608
Stomach	1.0 (ref)		1.0 (ref)	
Small intestine	2.54 (1.53–4.20)		1.43 (0.82–2.50)	
Colorectum/rectal Other	0.54 (0.07–3.98)		0.82 (0.11–6.22)	
	2.60 (1.05–6.39)		1.49 (0.58–3.83)	
CD34		0.362	-	
Negative	1.0 (ref)			
Positive	0.76 (0.43–1.37)			
CD117		0.238	-	
Negative	1.0 (ref)			
Positive	0.55 (0.20–1.49)			
DOG-1		<0.001		0.018
Negative	1.0 (ref)		1.0 (ref)	
Positive	0.29 (0.18–0.49)		0.52 (0.30–0.89)	
Gene mutation		0.990		
KIT exon 11	1.0 (ref)			
KIT exon 9	1.06 (0.61–1.82)			
PDGFR-α	1.14 (0.28–4.72)			
Wild type	1.22 (0.58–2.59)			
Other	0.00 (0.00–5.60)			
Neoadjuvant therapy with imatinib		0.280	-	
Not received	1.0 (ref)			
Received	0.46 (0.11–1.88)			
Adjuvant therapy with imatinib		<0.001		<0.001
Not received	1.0 (ref)		1.0 (ref)	
Drug discontinuance	0.28 (0.15–0.53)		0.33 (0.17–0.64)	
Drug continuance	0.22 (0.13–0.37)		0.19 (0.11–0.32)	

Note: “-” indicates no data. Abbreviation: RFS, recurrence-free survival; HR, hazard ratio; CI, confidence interval; DOG-1, gastrointestinal stromal tumor protein 1; CD117, cluster of differentiation 117; CD34, cell differentiation factor 34; HPF, high-power field.

**Figure 3:**
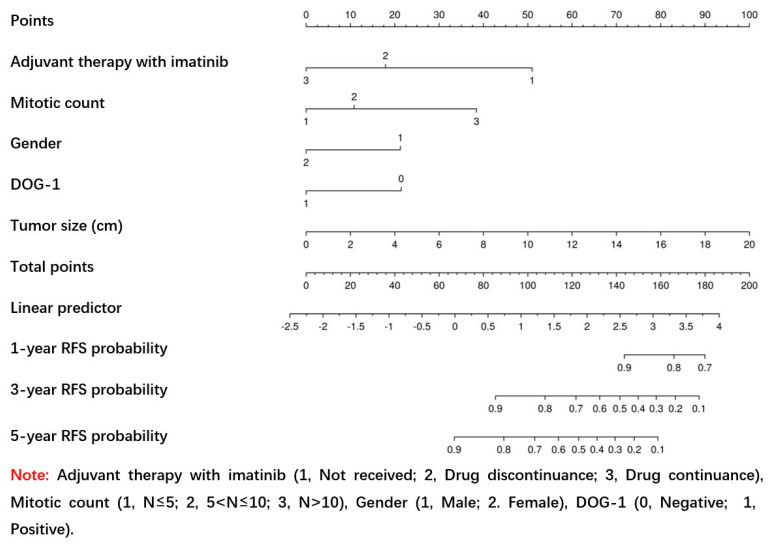
Nomogram predicting the probabilities of 1-, 3-, and 5-year RFS. Points are assigned for tumor size, gender, mitotic count, DOG-1, and adjuvant therapy with imatinib by drawing a line upward from the corresponding values to the “Points” line. The sum of these five points plotted on the “Total Points” line corresponds to predictions of 1-, 3-, and 5-year RFS.
